# Liquid biopsy using the supernatant of a pleural effusion for EGFR genotyping in pulmonary adenocarcinoma patients: a comparison between cell-free DNA and extracellular vesicle-derived DNA

**DOI:** 10.1186/s12885-018-5138-3

**Published:** 2018-12-10

**Authors:** Jong Sik Lee, Jae Young Hur, In Ae Kim, Hee Joung Kim, Chang Min Choi, Jae Chol Lee, Wan Seop Kim, Kye Young Lee

**Affiliations:** 1 Department of Pulmonary, Lung Cancer Center, Konkuk University Medical Center and Medicine, Konkuk University School of Medicine, 120-1 Hwayang-dong, Gwangjin-Gu, Seoul, 05030 Republic of Korea; 2 Department of Pulmonary Medicine, Konkuk University School of Medicine, Seoul, Republic of Korea; 3 Department of Pathology, Konkuk University School of Medicine, Seoul, Republic of Korea; 4 Department of Pulmonary and Critical Care Medicine, University of Ulsan, College of Medicine, Asan Medical Center, Seoul, Republic of Korea; 5 Department of Oncology, University of Ulsan, College of Medicine, Asan Medical Center, Seoul, Republic of Korea

**Keywords:** Pleural effusion, Pulmonary adenocarcinoma, EGFR mutation, Extracellular vesicles, Liquid biopsy

## Abstract

**Background:**

EGFR genotyping in pulmonary adenocarcinoma patients who develop pleural effusions is mostly performed using cytology or cell block slides with low sensitivity. Liquid biopsy using the supernatant of pleural effusions may be more effective because they contain many components released by cancer cells. Extracellular vesicles (EVs) are known to carry oncogenic double-stranded DNA that is considered a notable biomarker. Here, we investigate the efficiency of liquid biopsy using cell-free DNA (cfDNA) and extracellular vesicle-derived DNA (EV-derived DNA) from the supernatant of pleural effusions for EGFR genotyping in patients with pulmonary adenocarcinoma.

**Methods:**

Fifty pleural effusion samples from patients with pulmonary adenocarcinoma were evaluated. The supernatant, after removing the cell pellet by centrifugation, was used for liquid biopsy, and EVs were isolated from the pleural effusion by ultracentrifugation. EV-derived DNA and cfDNA were extracted separately, and EGFR genotyping was performed by the PNA clamping method.

**Results:**

Among 32 patients who were EGFR-tyrosine kinase inhibitor (TKI) naïve with a known tissue EGFR genotype, liquid biopsy using EV-derived DNA from the pleural effusion supernatant showed 100% matching results with tissue EGFR genotyping in 19 EGFR mutant cases and detected three additional EGFR mutations in patients with wild-type (WT) tissue. Liquid biopsy using cfDNA from pleural effusion supernatants missed two cases of tissue-based EGFR mutations and found two additional EGFR mutation cases. In 18 patients who acquired resistance to EGFR-TKI, EGFR genotyping using EV-derived DNA from the pleural effusion supernatant detected the T790 M mutation in 13 of 18 (72.2%) patients, and this mutation was detected in 11 (61.1%) patients using cfDNA. By contrast, only three patients were found to present the T790 M mutation when using cell block or cytology slides.

**Conclusions:**

Liquid biopsy using the supernatant of pleural effusions showed significantly improved results for EGFR genotyping compared to those using conventional cell block or cytology samples. Liquid biopsy using EV-derived DNA is promising for EGFR genotyping, including T790 M detection in pulmonary adenocarcinoma patients who develop pleural effusions.

**Electronic supplementary material:**

The online version of this article (10.1186/s12885-018-5138-3) contains supplementary material, which is available to authorized users.

## Background

Malignant pleural effusion is shown in 11–32% of patients with advanced non-small cell lung cancer (NSCLC) [1, 2]. The diagnostic rates of pleural effusion for malignancy have a mean sensitivity of approximately 60% (range 40–87%) [3]. Pleural effusion for adenocarcinoma gives higher diagnostic rates than those for squamous cell or small cell carcinomas. EGFR genotyping using malignant pleural effusion is useful when adequate tissue is not available due to co-morbidities or other reasons. In those cases, cell pellet specimens, including cell block or cytology, are conventionally used. EGFR mutation detection using malignant pleural effusion is less invasive and shows equivalent mutation detection rates when compared with a tissue biopsy [4]. Recently, supernatant samples known to be highly effective for EGFR genotyping have shown concordance with tissue typing [4, 5]. cfDNA is frequently used for liquid biopsy due to its convenience but has intrinsic problems of DNA stability and a short half-life (2 h) [6, 7].

Extracellular vesicles (EVs) are nanoparticles that contain bioactive molecules such as proteins and nucleic acids and have great potential as cancer biomarkers because the biomarkers are protected by a dual lipid membranous coat [8, 9]. EVs are released in large quantities in various biological fluids, such as pleural effusions, plasma, urine, saliva and bronchoalveolar lavage fluid [10]. Recently, it has been demonstrated in various cancer cell lines that the majority of DNA associated with tumor exosomes is double-stranded, which highlights the translational value of exosomal DNA for its potential usefulness as a circulating biomarker for cancer detection [9, 11, 12]. Furthermore, we demonstrated that EV-derived DNA is much more highly efficient for EGFR genotyping than cfDNA in lung cancer patients [13]. On this basis, we investigated the feasibility of EGFR genotyping using the supernatant of pleural effusions in comparison with tissue-based genotyping in patients with pulmonary adenocarcinoma. We also compared the translational value of the cfDNA and EV-derived DNA extracted from the supernatant of pleural effusions for EGFR genotyping in EGFR-TKI-naïve patients and for detecting the T790 M mutation in patients with acquired resistance to EGFR-TKIs.

## Methods

### Study population and pleural effusion samples

Among patients diagnosed and treated for pulmonary adenocarcinoma with pleural effusion at Konkuk University Medical Center and Asan Medical Center between January 1, 2011 and December 31, 2015, fifty patients with archival pleural effusion samples were included in the analysis. Demographics and clinical data of the included patients were reviewed. This study was conducted in accordance with the amended Declaration of Helsinki. The study protocol was approved by the institutional review board of Konkuk University Medical Center and Asan Medical Center, and written informed consent was obtained from all patients. After 50 mL of a pleural effusion sample was centrifuged using Falcon™ 50 mL conical centrifuge tubes at 1000 g for 10 min at 4 °C to remove cells and debris, the supernatant of the pleural effusion was separated and stored at − 80 °C until further evaluation.

### Extracellular vesicles (EVs) isolation and characterization

Supernatant samples of the pleural effusions were each transferred to an ultracentrifuge tube and spun at 200,000 g for 1 h at 4 °C. The volume of sample used for the isolation of EVs was 500 μL of pleural effusion supernatant. The supernatant was carefully removed, and the pellet was resuspended in 200 μL of PBS for DNA extraction. We eluted EV-derived DNA with 50 μL of distilled water. The purified EV size was analyzed using NanoSight.

### Extraction of EV-derived DNA and cfDNA

To eliminate free-floating DNA that exists outside of EVs, purified EVs were added to a microtube. Then, the EVs were treated with 10x reaction buffer (200 mM Tris-HCl, pH 8.3, 20 mM MgCl2) and DNase I (Sigma, St. Louis, MO) and then incubated for 15 min at room temperature. To bind calcium and magnesium ions and to inactivate the DNase I, the sample was treated with stop solution (50 mM EDTA), and the microtube was heated at 70 °C for 10 min to denature the DNase I and then chilled on ice. After elimination of free-floating DNA, the EVs were lysed by mixing cell lysis buffer and detergent, and the EV-derived DNA was purified using the High Pure PCR Template Preparation Kit (Roche Diagnostics, Mannheim, Germany). The volume of sample used for the isolation of cfDNA was 500 μL of pleural effusion supernatant. cfDNA was isolated from the supernatant of the pleural effusion using the High Pure PCR Template Preparation Kit (Roche Diagnostics, Mannheim, Germany). We eluted cfDNA with 50 μL of distilled water. The purification of DNA was measured using a NanoDrop Spectrophotometer (Thermo Fisher Scientific, Waltham, MA USA). The concentration and distribution of double-strand DNA was measured using a 4200 TapeStation (Agilent Technologies, Santa Clara, CA, USA).

### EGFR genotyping

The EGFR genotyping was tested using the PNAClamp™ EGFR Mutation Detection Kit (Panagene, Daejeon, Korea) with the peptide nucleic acid (PNA)-mediated PCR clamping method. Each sample was tested in eight individual reaction tubes for 29 EGFR DNA mutations. All reactions had a total reaction volume of 20 μL, which contained 70 ng of template DNA, a primer, a PNA probe set, and a fluorescence dye PCR master mix. All reagents were included with the kit. PNA-mediated clamping PCR reactions were performed using a CFX96TM (Bio-Rad, Hercules, CA). The PCR cycling conditions were as follows: 5 min at 94 °C followed by 40 cycles at 94 °C for 30 s, 70 °C for 20 s, 63 °C for 30 s, and 72 °C for 30 s. The efficiency of PCR clamping was determined by measuring the threshold cycle (Ct) value. Ct values for the control and mutation assays were obtained from the fluorescence amplification plots. The delta Ct (ΔCt) value was as follows: ΔCt-1 = [standard Ct − sample Ct]. The cut-off ΔCt-1 was defined as 2.0 for the 29 mutations in EGFR DNA [14].

### Statistical analysis

Data were summarized as medians with interquartile range (IQR) with non-normal distribution. We used the Wilcoxon signed rank test to test the significance of differences in DNA concentration of EV-derived and cfDNA. The EGFR genotype agreement between the tissue and EV-derived or cfDNA was measured by Cohen’s kappa coefficient. All statistical analyses were carried out using SPSS Statistics version 24 (IBM Corp, Chicago, IL, USA), and a *P* value < 0.05 was regarded as statistically significant.

## Results

### Characteristics of patients

We analyzed 50 pleural effusion samples that were obtained from patients with pulmonary adenocarcinoma. The median age of all patients was 67.5 years (IQR 59.8–73.3), and 25 (50%) were female. Twenty-five (50%) patients were non-smokers; 14 (28%) were ex-smokers, and 11 (22%) were current smokers. EGFR genotyping based on tumor tissue showed 19 deletions in 22 (44%) patients, L858R in 15 (30%), and wild-type EGFR in 13 (26%). Of the 50 patients, 32 were EGFR-TKI naïve and presented pleural effusion at the time of diagnosis or after treatment failure. Eighteen patients displayed acquired resistance to EGFR-TKI, who developed pleural effusion after TKI treatment (Table 1).

**Table 1:** Clinical characteristics of the patients

Patient classification	All patients (*N* = 50)	EGFR-TKI naïve patients (*N* = 32)	EGFR-TKI acquired-resistance patients (*N* = 18)
Age, year, median (IQR)	67.5 (59.8–73.3)	68.5 (58.3–73.8)	67.5 (62.7–73.5)
Sex, female	25 (50.0%)	14 (43.8%)	11 (61.1%)
Smoking status			
Non-smoker	25 (50.0%)	13 (40.6%)	12 (66.7%)
Ex-smoker	14 (28.0%)	9 (28.1%)	5 (27.8%)
Current smoker	11 (22.0%)	10 (31.3%)	1 (5.6%)
Tumor EGFR mutation			
Exon 19 deletion	22 (44.0%)	10 (31.3%)	12 (66.7%)
L858R	15 (30.0%)	9 (28.1%)	6 (33.3%)
Wild-type	13 (26.0%)	13 (40.6%)	0
Histology			
Adenocarcinoma	50	32	18

*EGFR-TKI* epidermal growth factor receptor tyrosine kinase inhibitor; *IQR* interquartile range

### Characterization of isolated EVs from the supernatant of pleural effusions

The size and concentration of EVs isolated from malignant pleural effusion and parapneumonic effusion were analyzed. Isolated EVs from the supernatant of the pleural effusion of an EGFR-TKI naïve patient (case 5) showed a mean size of 267.6 nm and concentration of 4.18 × 10^8^ particles/mL, but isolated EVs from the supernatant of parapneumonic effusion of a patient with bacterial pneumonia displayed a mean size of 174.3 nm and concentration of 1.10 × 10^9^ particles/mL. When comparing between malignant pleural effusion and parapneumonic effusion, the concentration of EVs isolated from parapneumonic effusion was higher than that from malignant effusion, and by contrast, the mean size of EVs isolated from malignant pleural effusion was larger than the mean size of those from isolated from parapneumonic effusion. Malignant pleural effusion exhibited bimodal EV distributions (Additional file 1: Figure S1A, B) [15, 16].

### Quantitative and qualitative characterization of EV-derived DNA and cfDNA isolated from the supernatant of pleural effusions

EV-derived DNA and cfDNA were extracted from the supernatant of pleural effusions from patients who were EGFR-TKI naïve (case 9). TapeStation analysis showed that the concentration of EV-derived DNA from the supernatant of the pleural effusion was 3.18 ng/μL, which was higher than the concentration of cfDNA (2.31 ng/μL). When comparing the distributions of double-stranded DNA (dsDNA) size between EV-derived DNA and cfDNA from the supernatant of a pleural effusion across all DNA size scales, EV-derived DNA from the supernatant of the pleural effusion showed higher intensity. cfDNA had a higher fraction of 600 bp DNA fragments, displaying a noticeable peak around that size, as EV-derived DNA did not show this peak. When compared with cfDNA, EV-derived DNA contained more diverse-sized dsDNA fragments (Additional file 1: Figure S1C). Such difference between cfDNA and EV-derived DNA seems to arise when cfDNA is extracted from pleural effusions due to various matters such as small size of EV and lipoproteins that prevent lysis of EV lipid membrane, which leads to rare EV-derived DNA in the extracts. That is why EV is isolated from pleural effusions before DNA is extracted.

### Agreement of EGFR genotyping using EV-derived DNA and cfDNA extracted from the supernatant of pleural effusions in EGFR-TKI naïve patients

**Fig. 1:**
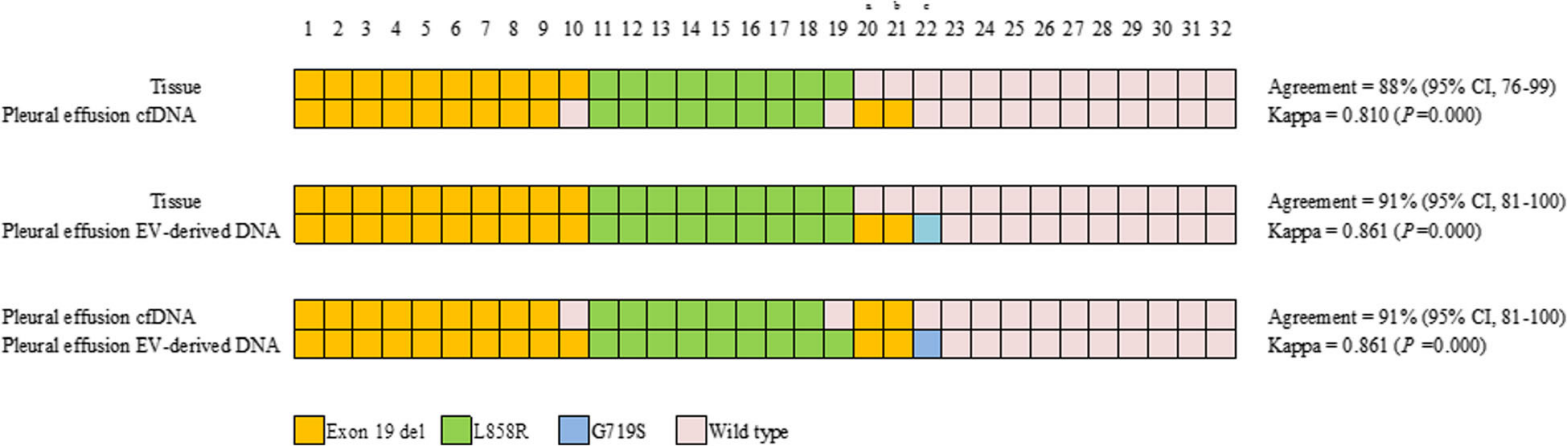
Agreement of EGFR genotyping in cell-free and EV-derived DNA from the supernatant of pleural effusions compared with tumor tissue from EGFR-TKI naïve patients. cfDNA = cell-free DNA; EV-derived DNA = extracellular vesicle-derived DNA; a, c = follow-up loss after diagnosis; b = TKI response could not be evaluated as the patient had expired during TKI treatment

Out of 32 patients who were EGFR-TKI naïve, tissue EGFR genotyping results revealed 19 EGFR mutant cases (10 in Exon 19del and 9 L858R) and 13 wild-type cases. EGFR genotyping using EV-derived DNA from the supernatant of the pleural effusions showed perfect agreement (19/19) with tissue EGFR genotyping across the 19 EGFR mutant cases, and this method identified three additional EGFR mutations (cases 20, 21 and 22) in cases in which tissue EGFR genotyping showed wild-type results. The percent agreement between EV-derived DNA and tissue genotyping was 91% (κ = 0.861; *p* = 0.000) (Fig. 1, Additional file 2: Table S1). Since two patients (cases 20 and 22) did not undergo follow-up after lung cancer diagnosis, and one patient (case 21) died during EGFR-TKI treatment, tumor response to EGFR-TKI in the three additional EGFR mutant cases could not be verified. Compared with tissue EGFR genotyping for EGFR mutant cases, EGFR genotyping using cfDNA from the supernatant of pleural effusion detected 17 of the 19 (89%) mutant cases. Although two patients (cases 10 and 19) were missing among the 19 mutant cases, two additional mutant cases (cases 20 and 21) were found among wild-type EGFR tissue cases. In EGFR genotyping using cfDNA, 88% of cases were concordant with tissue EGFR genotyping (κ = 0.810; *p* = 0.000) (Fig. 1, Additional file 2: Table S1).

### T790 M detection using EV-derived DNA and cfDNA from the supernatant of pleural effusions in patients with acquired resistance to EGFR-TKI

**Fig. 2:**
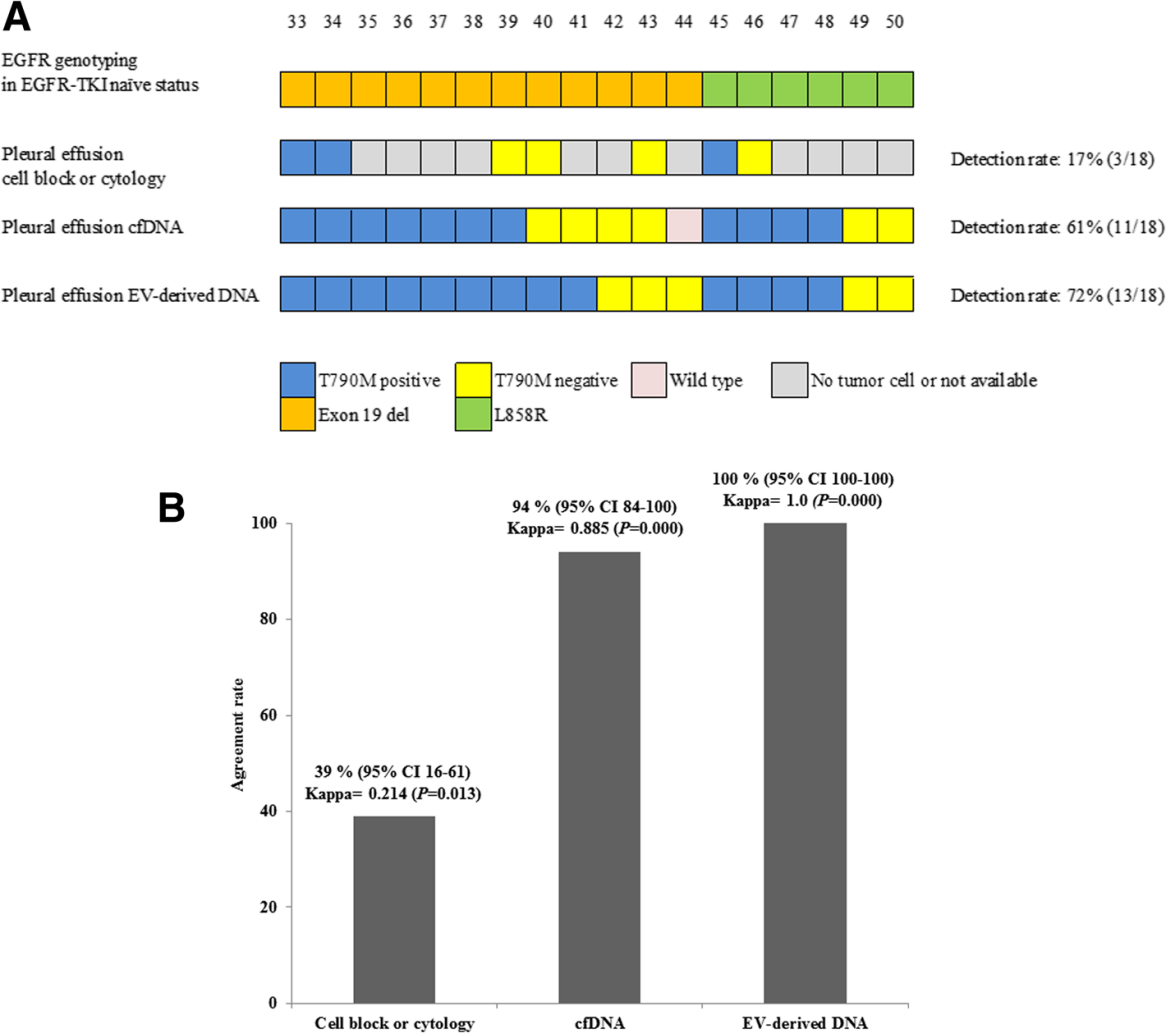
Comparison of the EGFR genotyping between cell block or cytology, cell-free DNA and EV-derived DNA from the supernatant of pleural effusions in EGFR-TKI acquired-resistance patients. **a** Detection of the T790 M mutation using cell block or cytology, cfDNA and EV-derived DNA of pleural effusions. The detection rate of the T790 M mutation is 17, 61, 72% in cell block or cytology, cfDNA, and EV-derived DNA of pleural effusions, respectively. **b** EGFR genotyping in accordance with tissue using a cell block or cytology, cfDNA and EV-derived DNA of pleural effusion are 39, 94, and 100%, respectively. cfDNA = cell-free DNA; EV-derived DNA = extracellular vesicle-derived DNA

T790 M detection was examined in 18 patients who manifested with pleural effusion after TKI treatment. Among the 18 patients, tissue EGFR genotyping showed Exon 19del in 12 patients and L858R in 6 patients. EGFR genotyping using EV-derived DNA from the supernatant of the pleural effusions detected T790 M in 13 of the 18 (72%) patients (nine patients with T790 M + Exon 19 del and four patients with T790 M + L858R). EGFR genotyping using cfDNA from the supernatant of the pleural effusions found T790 M in 11 of 18 (61%) patients (seven patients with T790 M + Exon 19del and four patients with T790 M + L858R). When using EV-derived DNA from the supernatant of the pleural effusions for EGFR genotyping, two more T790 M mutations were detected in comparison with using cfDNA from the supernatant of the pleural effusions. By the conventional cell block or cytology slides which were made from the stored pleural effusion samples, because of not available tissue rebiopsy, only three patients were identified as T790 M positive (two patients with T790 M + Exon 19 del and one patient with T790 M + L858R). We identified five out of nine patients who showed no tumor results from cell block or cytology samples that have the T790 M mutation when tested with EV-derived DNA (Fig. 2a, Additional file 3: Table S2). Furthermore, EGFR genotyping using EV-derived DNA from the supernatant of pleural effusions showed 100% agreement with tissue EGFR genotyping (κ = 1.0; *p* = 0.000). When using cfDNA from the supernatant of pleural effusions for EGFR genotyping, 17 out of 18 patients displayed agreement with the tissue EGFR genotyping, whereas one patient did not exhibit agreement (94% agreement, κ = 0.885; *p* = 0.000). When using a cell block or cytology, 39% of the results were concordant with tissue EGFR genotyping (κ = 0.214; *p* = 0.013) (Fig. 2b, Additional file 3: Table S2).

### Comparison of the DNA concentration of EV-derived DNA and cfDNA from the supernatant of pleural effusions

From 32 patients who were EGFR-TKI naïve and 18 who acquired EGFR-TKI resistance, the concentrations of EV-derived DNA and cfDNA from the supernatant of pleural effusions were measured by NanoDrop spectrophotometry. Overall, the DNA concentration of EV-derived DNA was higher than that of cfDNA (Additional file 2: Table S1, Additional file 3: Table S2). The median concentrations of EV-derived DNA and cfDNA were 22.7 (15.7–36.2) vs 14.7 (10.5–24.3) for patients who were EGFR-TKI naïve (*p* = 0.000) and 18.2 (14.5–32.1) vs 15.4 (11.1–21.8) for patients with acquired resistant to EGFR-TKI (*p* = 0.001). The DNA concentration of EV-derived DNA from the supernatant of pleural effusions was significantly higher than that of cfDNA (Table 2).

**Table 2:** Concentrations of cfDNA and EV-derived DNA from the supernatant of the pleural effusions

		Supernatant of pleural effusion		
		cfDNA concentration (ng/μL)	EV-derived DNA concentration (ng/μL)	*P* value
EGFR-TKI naïve patients	*N* = 32	14.7 (10.5–24.3)	22.7 (15.7–36.2)	0.000
EGFR-TKI acquired resistance patients	*N* = 18	15.4 (11.1–21.8)	18.2 (14.5–32.1)	0.001

Data are expressed as median (IQR), *cfDNA* cell-free DNA, *EV-derived DNA* extracellular vesicle-derived DNA

## Discussion

In general, tumor tissue obtained by surgery or biopsy has been used for EGFR genotyping in patients with NSCLC, among whom 27–31% of biopsies may fail to acquire tumor tissue that is suitable for EGFR genotyping at diagnosis or during disease progression [17]. When tumor tissue is not accessible, plasma cfDNA provides a promising diagnostic approach for detecting EGFR mutations and has been shown to be effective for diagnosis in meta-analysis [18, 19]. Previous studies into the detection of EGFR mutations using plasma cfDNA have noted low sensitivity compared with tumor tissue biopsies with an average sensitivity of 65–70% and high specificity (> 88%) [20, 21]. For detecting T790 M among patients with NSCLC with acquired resistance, when repeated biopsy, circulating tumor cell (CTC) DNA, and circulating tumor DNA (ctDNA) were combined, T790 M was detected in 73% of patients [22]. The combination of CTC and ctDNA genotyping allowed for the identification of the T790 M mutation in 14 (35%) patients for whom the concurrent biopsy was either indeterminate or T790 M negative [22].

Recent studies have shown that the cell-free supernatant of pleural effusion might be a better resource for mutation detection than cell pellets [4], and EGFR mutations have been identified in supernatant samples [5]. In our study, EGFR genotyping using EV-derived DNA and cfDNA from the supernatant of pleural effusions in patients who were either EGFR-TKI naïve or who acquired resistance to EGFR-TKI showed good agreement with tissue EGFR genotyping (κ =0.861, 0.810, 1.0, 0.885). Furthermore, the detection rate of T790 M using EV-derived DNA from the supernatant of pleural effusions is more efficient than those using cell blocks or cfDNA.

Cancer cell-derived EVs might manifest the content of their cells of origin and maintain the potential to transfer specific oncogenic components to recipient cells [23]. Furthermore, several studies have also identified double-stranded DNA fragments in the EVs ranging from 100 b to 17 kb in size [11, 12]. In the supernatants of pancreatic cancer cells and serum from patients with pancreatic cancer, Sanger sequencing analyses of polymerase chain reaction (PCR) amplicons have identified KRAS and TP53 mutations [11]. Since EVs are stable sources of genetic and protein cargos from the cell of origin, EVs are interesting as cancer biomarkers. The extensively fragmented nature of circulating cfDNA makes it difficult for this format to become generalizable in the context of genomic characterization through current next-generation sequencing (NGS) platforms [24]. By contrast, EV-derived DNA is long-stranded, has a high concentration, and would be easy to amplify, which means it could therefore be a candidate for NGS approaches. Previous studies have displayed a sensitivity of approximately 70% for EGFR genotyping by digital PCR [25], and our study demonstrated high sensitivity using a commonly available PNA clamping method.

In this study of pulmonary adenocarcinoma patients with pleural effusions that developed before or after EGFR-TKI treatment, we identified several interesting findings. First, in patients who were EGFR-TKI naïve, EGFR genotyping using EV-derived DNA from the supernatant of pleural effusions is in 100% agreement with tissue EGFR genotyping and has higher sensitivity, as this method was capable of detecting three additional mutant cases in patients who were otherwise diagnosed as tissue wild-type. We observed that EGFR genotyping using cfDNA from the supernatant of pleural effusions missed two tissue-proven EGFR mutations but detected two more mutant cases in patients diagnosed as tissue wild-type. Second, for detecting T790 M in patients with acquired resistance to EGFR-TKI with pleural effusion, using the supernatant of the pleural effusion to acquire EV-derived and cfDNA was significantly superior to the conventional use of cell pellet methodologies, such as cytology or using cell block specimens. Third, EV-derived DNA from the supernatant of pleural effusions outperformed cfDNA from pleural effusions for the EGFR genotyping of patients with pulmonary adenocarcinoma with pleural effusion.

Tumor necrosis in malignant pleural effusions produces a spectrum of DNA fragments with variable strand lengths due to random and incomplete genomic DNA digestion by deoxyribonucleases, most of which are long DNA fragments. It has been suggested that tumor cells may actively secrete DNA fragments, as has been observed for lymphocytes, extracellular vesicles of NSCLC and cancer cell lines [26–28]. The supernatant of pleural effusions might have high-quality DNA that could be amplifiable in the form of EV-derived DNA and cfDNA.

This study has several limitations. First, the number of patients included in the analysis was small, and only archival pleural effusions were examined. To confirm our observations, a large-scale study enrolling patients from multiple hospitals is needed. Second, this study was performed retrospectively, and the time points of pleural effusion analysis were not strictly controlled. Additionally, crucial data, such as the cell block or cytology of the pleural effusions, were missing for some patients. Third, third-generation EGFR-TKIs were not available in Korea until a few years ago. Therefore, tumor response to third-generation EGFR-TKIs was not evaluated. Further studies will be required to correlate the clinical response with EV-derived DNA and cfDNA genotyping from the supernatant of pleural effusions.

## Conclusions

In conclusion, the supernatant of pleural effusions is particularly more effective for EGFR genotyping than conventional cytology or cell block samples for patients with pulmonary adenocarcinoma. EV-derived DNA from the supernatant of pleural effusions is promising for EGFR genotyping including T790 M detection in pulmonary adenocarcinoma patients who develop pleural effusion.

## Additional files

Additional file 1: **Figure S1.** (A, B) EV quantification using NanoSight (Nanoparticle tracking analysis). EVs from the supernatant of malignant pleural effusions and parapneumonic effusions show mean sizes of 267.6 of 174.3 nm and concentrations 4.18 × 10^8^ and 1.10 × 10^9^ particles/ml, respectively. (C) EVs derived from the supernatant of malignant pleural effusions show a higher double-strand DNA concentration compared to that of cfDNA, as assessed by a microfluidic platform based on an electrophoretic system (4200 TapeStation, Agilent). (TIF 64 kb)

Additional file 2: **Table S1.** Comparison of the EGFR genotyping between tumor tissue, cell-free DNA and EV-derived DNA from the supernatant of pleural effusions in EGFR-TKI naïve patients. (DOCX 19 kb)

Additional file 3: **Table S2.** DNA concentration of cell-free DNA and EV-derived DNA from the supernatant of pleural effusions in EGFR-TKI acquired-resistance patients. (DOCX 21 kb)

## Abbreviations

cf: Cell-free
EGFR: Epidermal growth factor receptor
EV: Extracellular vesicle
IQR: Interquartile range
NGS: Next-generation sequencing
NSCLC: Non-small cell lung cancer
PCR: Polymerase chain reaction
PNA: Peptide nucleic acid
TKI: Tyrosine kinase inhibitor
WT: Wild-type
