# Supplementary figures and images for: Liquid biopsy using the supernatant of a pleural effusion for EGFR genotyping in pulmonary adenocarcinoma patients: a comparison between cell-free DNA and extracellular vesicle-derived DNA
Source: BMC Cancer. 2018 Dec 10;18:1236. doi: 10.1186/s12885-018-5138-3 (PMC6288853; doi:10.1186/s12885-018-5138-3)

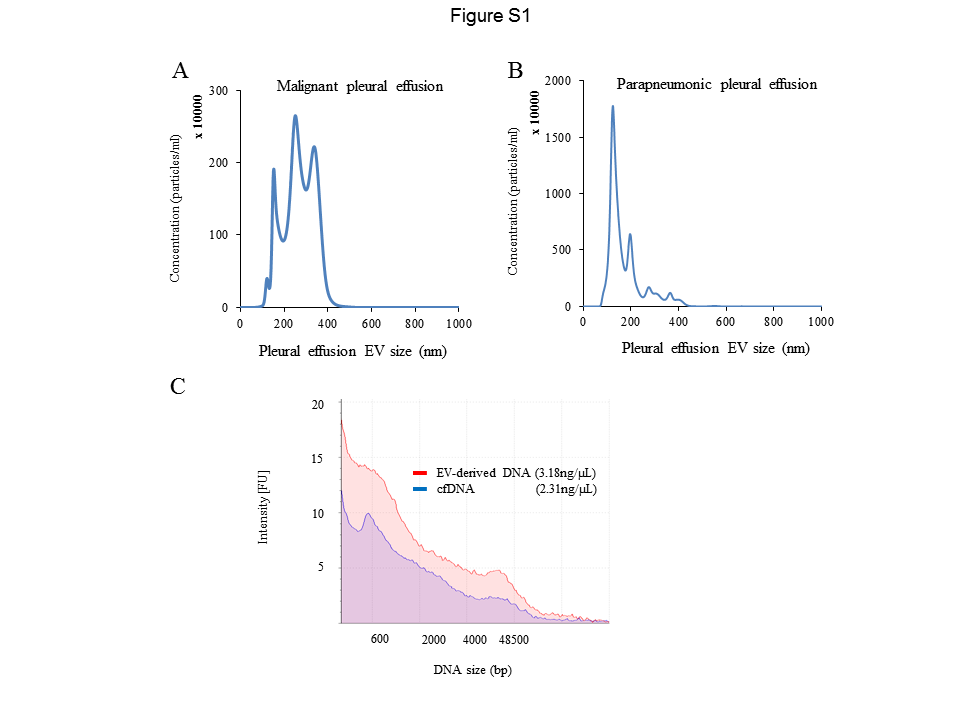

Supplement: Supplementary file 1 — Figure S1. (A, B) EV quantification using NanoSight (Nanoparticle tracking analysis). EVs from the supernatant of malignant pleural effusions and parapneumonic effusions show mean sizes of 267.6 of 174.3 nm and concentrations 4.18 × 10^8^ and 1.10 × 10^9^ particles/ml, respectively. (C) EVs derived from the supernatant of malignant pleural effusions show a higher double-strand DNA concentration compared to that of cfDNA, as assessed by a microfluidic platform based on an electrophoretic system (4200 TapeStation, Agilent). (TIF 64 kb) [file 12885_2018_5138_MOESM1_ESM.tif]
