# Supplementary material for: Liquid biopsy using the supernatant of a pleural effusion for EGFR genotyping in pulmonary adenocarcinoma patients: a comparison between cell-free DNA and extracellular vesicle-derived DNA
Source: BMC Cancer. 2018 Dec 10;18:1236. doi: 10.1186/s12885-018-5138-3 (PMC6288853; doi:10.1186/s12885-018-5138-3)
Supplement: Supplementary file 2 — Table S1. Comparison of the EGFR genotyping between tumor tissue, cell-free DNA and EV-derived DNA from the supernatant of pleural effusions in EGFR-TKI naïve patients. (DOCX 19 kb) [file 12885_2018_5138_MOESM2_ESM.docx]

Additional file 2: Table S1. Comparison of the EGFR genotyping between tumor tissue, cell-free DNA and EV-derived DNA from the supernatant of pleural effusions in EGFR-TKI naïve patients.

| No. | Tumor tissue | Supernatant of pleural effusion | | | | |
| --- | --- | --- | --- | --- | --- | --- |
|  |  | Cell-free DNA | DNA concentration (ng/µL) | EV-derived DNA | DNA concentration (ng/µL) | Sampling time |
| 1 | Exon 19 del | Exon 19 del | 14.5 | Exon 19 del | 25.7 | Treatment-naïve |
| 2 | Exon 19 del | Exon 19 del | 19.08 | Exon 19 del | 18.82 | Treatment-naïve |
| 3 | Exon 19 del | Exon 19 del | 21.23 | Exon 19 del | 20.65 | Treatment-naïve |
| 4 | Exon 19 del | Exon 19 del | 14.65 | Exon 19 del | 14.88 | Treatment-naïve |
| 5 | Exon 19 del | Exon 19 del | 12.9 | Exon 19 del | 12.9 | Treatment-naïve |
| 6 | Exon 19 del | Exon 19 del | 40.7 | Exon 19 del | 86.4 | Treatment-naïve |
| 7 | Exon 19 del | Exon 19 del | 24.1 | Exon 19 del | 25.1 | Treatment-naïve |
| 8 | Exon 19 del | Exon 19 del | 75.5 | Exon 19 del | 116.74 | Treatment-naïve |
| 9 | Exon 19 del | Exon 19 del | 4.9 | Exon 19 del | 22.7 | Treatment-naïve |
| 10 | Exon 19 del | WT | 41.9 | Exon 19 del | 30.7 | Treatment-naïve |
| 11 | L858R | L858R | 28.2 | L858R | 57.5 | Treatment-naïve |
| 12 | L858R | L858R | 13.9 | L858R | 15.7 | Treatment-naïve |
| 13 | L858R | L858R | 28.4 | L858R | 37.3 | Treatment-naïve |
| 14 | L858R | L858R | 14.71 | L858R | 41.67 | Treatment-naïve |
| 15 | L858R | L858R | 19.69 | L858R | 30.31 | Treatment-naïve |
| 16 | L858R | L858R | 24.42 | L858R | 33.04 | Treatment-naïve |
| 17 | L858R | L858R | 13.8 | L858R | 40.1 | Treatment-naïve |
| 18 | L858R | L858R | 16.95 | L858R | 22.28 | Treatment-naïve |
| 19 | L858R | WT | 3.8 | L858R | 14 | Recurrence after surgery |
| 20^a^ | WT | Exon 19 del | 12.97 | Exon 19 del | 15.12 | Treatment-naïve |
| 21^b^ | WT | Exon 19 del | 14.72 | Exon 19 del | 16.27 | After 1^st^ line treatment failure |
| 22^c^ | WT | WT | 3.3 | G719S | 17.9 | Treatment-naïve |
| 23 | WT | WT | 10.48 | WT | 15.74 | Treatment-naïve |
| 24 | WT | WT | 16.95 | WT | 22.28 | After 1^st^ line treatment failure |
| 25 | WT | WT | 7.5 | WT | 25.9 | Treatment-naïve |
| 26 | WT | WT | 2.2 | WT | 13.7 | Treatment-naïve |
| 27 | WT | WT | 4.3 | WT | 27.6 | Treatment-naïve |
| 28 | WT | WT | 13.33 | WT | 14.89 | After 1^st^ line treatment failure |
| 29 | WT | WT | 10.59 | WT | 22.73 | Treatment-naïve |
| 30 | WT | WT | 6.6 | WT | 12.92 | Treatment-naïve |
| 31 | WT | WT | 61.3 | WT | 225.5 | Treatment-naïve |
| 32 | WT | WT | 59 | WT | 56 | Treatment-naïve |

del= deletion; a,c= follow-up loss after diagnosis; b= TKI response could not be evaluated because the patient was expired during TKI treatment.
