# Supplementary material for: Liquid biopsy using the supernatant of a pleural effusion for EGFR genotyping in pulmonary adenocarcinoma patients: a comparison between cell-free DNA and extracellular vesicle-derived DNA
Source: BMC Cancer. 2018 Dec 10;18:1236. doi: 10.1186/s12885-018-5138-3 (PMC6288853; doi:10.1186/s12885-018-5138-3)
Supplement: Supplementary file 3 — Table S2. DNA concentration of cell-free DNA and EV-derived DNA from the supernatant of pleural effusions in EGFR-TKI acquired-resistance patients. (DOCX 21 kb) [file 12885_2018_5138_MOESM3_ESM.docx]

| No. | Tumor tissue | Cell block or Cytology | Supernatant of pleural effusion | | | | | |
| --- | --- | --- | --- | --- | --- | --- | --- | --- |
|  |  |  | Cell-free DNA | DNA concentration, (ng/µL) | EV-derived DNA | DNA concentration, (ng/µL) | Months after start of EGFR-TKI therapy | Sampling time |
| 33 | Exon 19 del | Exon 19 del + T790M | Exon 19 del + T790M | 15.19 | Exon 19 del + T790M | 15.50 | 29 mon | During 4^th^ line Chemotherapy |
| 34 | Exon 19 del | Exon 19 del + T790M | Exon 19 del + T790M | 10.70 | Exon 19 del + T790M | 12.70 | 29 mon | During 1^st^ line  EGFR-TKI therapy |
| 35 | Exon 19 del | No tumor cell | Exon 19 del + T790M | 10.09 | Exon 19 del + T790M | 12.61 | 8 mon | During 2^nd^ line chemotherapy |
| 36 | Exon 19 del | No tumor cell | Exon 19 del + T790M | 20.49 | Exon 19 del + T790M | 31.20 | 28 mon | During 6^th^ line chemotherapy |
| 37 | Exon 19 del | No tumor cell | Exon 19 del + T790M | 18.00 | Exon 19 del + T790M | 14.50 | 5 mon | During 1^st^ line  EGFR-TKI therapy |
| 38 | Exon 19 del | No tumor cell | Exon 19 del + T790M | 16.84 | Exon 19 del + T790M | 22.22 | 28 mon | During 4^th^ line Chemotherapy |
| 39 | Exon 19 del | Exon 19 del | Exon 19 del + T790M | 36.12 | Exon 19 del + T790M | 49.00 | 11mon | During 1^st^ line  EGFR-TKI therapy |
| 40 | Exon 19 del | Exon 19 del | Exon 19 del | 29.95 | Exon 19 del + T790M | 30.70 | 6 mon | During 1^st^ line  EGFR-TKI therapy |
| 41 | Exon 19 del | Not available | Exon 19 del | 10.20 | Exon 19 del + T790M | 14.35 | 15 mon | During 1^st^ line  EGFR-TKI therapy |
| 42 | Exon 19 del | No tumor cell | Exon 19 del | 12.30 | Exon 19 del | 16.20 | 5 mon | During 1^st^ line  EGFR-TKI therapy |
| 43 | Exon 19 del | Exon 19 del | Exon 19 del | 15.60 | Exon 19 del | 13.80 | 21 mon | During 1^st^ line  EGFR-TKI therapy |
| 44 | Exon 19 del | No tumor cell | WT | 12.94 | Exon 19 del | 18.46 | 11 mon | During 1^st^ line  EGFR-TKI therapy |
| 45 | L858R | L858R + T790M | L858R + T790M | 3.40 | L858R + T790M | 16.40 | 26 mon | During 4^th^ line Chemotherapy |
| 46 | L858R | L858R | L858R + T790M | 41.19 | L858R + T790M | 63.89 | 9 mon | During 1^st^ line  EGFR-TKI therapy |
| 47 | L858R | No tumor cell | L858R + T790M | 17.64 | L858R + T790M | 27.59 | 19 mon | During 3^th^ line Chemotherapy |
| 48 | L858R | Not available | L858R + T790M | 11.20 | L858R + T790M | 34.88 | 29 mon | During 2^nd^ line Chemotherapy |
| 49 | L858R | No tumor cell | L858R | 14.62 | L858R | 17.93 | 12 mon | During 2^nd^ line Chemotherapy |
| 50 | L858R | No tumor cell | L858R | 25.80 | L858R | 37.00 | 2 mon | During 1^st^ line  EGFR-TKI therapy |

Additional file 3: Table S2. DNA concentration of cell-free DNA and EV-derived DNA from the supernatant of pleural effusions in EGFR-TKI acquired-resistance patients.
